# Effect of saliva from horse fly *Hybomitra bimaculata* on kinetic properties of Na,K-ATPase: possible role in regulation of relaxation

**DOI:** 10.2478/v10102-011-0024-8

**Published:** 2011-09

**Authors:** Katarína Wachalová, Jana Vlkovicová, Veronika Javorková, Lucia Mézešová, Peter Takác, Milan Kozánek, Milan Labuda, Patricia A. Nuttall, Norbert Vrbjar

**Affiliations:** 1 Institute for Heart Research, Department of Biochemistry, Slovak Academy of Sciences, Bratislava, Slovak Republic; 2 Institute of Zoology, Slovak Academy of Sciences, Bratislava, Slovak Republic; 3 Centre of Ecology and Hydrology NERC, Oxford, United Kingdom

**Keywords:** sodium pump, heart, horse fly, salivary glands

## Abstract

The possible involvement of salivary gland extract (SGE) from horse flies in modifying hyperpolarization and relaxation via alterations in functional properties of sarcolemmal Na,K-ATPase in the host tissue was tested *in vitro* by application of various amounts of SGE from *Hybomitra bimaculata*.

SGE in the amount of 3 µg proteins representing approximately the equivalent of one salivary gland of *Hybomitra bimaculata* induced a stimulatory effect on Na,K-ATPase at all ATP concentrations applied. This effect resulted from the improved ATP-binding site affinity in the Na,K-ATPase molecule, as implicated by the reduction in K_M_. Increasing the amount of SGE to 6.5 µg resulted in inhibition of the enzyme, which was characterized by reduction in V_max_ and also K_M_. This suggests that in the presence of relatively high *Hybomitra bimaculata* SGE concentration some SGE components affect Na,K-ATPase, when ATP is already bound to the enzyme.

Our results indicate that SGE from the horse fly *Hybomitra bimaculata* contain at least two different biologically active compounds modifying the acute recovery and maintenance of excitability during contractile activity in the host tissue by affecting Na,K-ATPase with opposite effects, depending on the ratio of SGE-proteins to proteins of the host tissue.

## Introduction

Sodium-potassium-activated ATPase (Na,K-ATPase or sodium pump, E.C. 3.6.3.9) is an integral membrane enzyme that pumps intracellular Na^+^ and extracellular K^+^ inward (Blanco & Mercer, 1998). The enzyme is involved in several cellular functions, such as maintenance of intracellular ionic concentrations, creation of transmembrane potential, regulation of cell volume, growth and differentiation, as well as regulation of contraction-relaxation processes in muscle cells (Ewart & Klip, 1995). Na,K-ATPase has been implicated in the mechanism of relaxation in vascular smooth muscle cells (Somlyo *et al.*, 1970; Hamlyn *et al.*, 1982; Overbeck *et al.*, 1987) and also in cardiac muscle cells (Deleze, 1960; Dhalla *et al.*, 1977; Glitsch, 2001).

A variety of hormones (insulin, insulin-like growth factor I, adrenaline, noradrenaline, calcitonin gene-related peptide, calcitonin, amylin) increase the rate of active Na^+^, K^+^ transport by 60–120% within a few minutes. This leads to a decrease in intracellular Na^+^ and hyperpolarization. In isolated muscles, where contractility is inhibited by high extracellular K^+^, such agents produce rapid force recovery which is entirely suppressed by ouabain and closely correlated to the stimulation of K^+^ uptake and the decline in intracellular Na^+^. The observations support the conclusion that Na,K-ATPase plays a central role in acute recovery and maintenance of excitability during contractile activity (Clausen, 1996).

Previously it was shown that relaxation of rat femoral artery was stimulated in a dose dependent manner by salivary gland extract (SGE) from horse flies Hybomitra bimaculata (Rajská *et al.*, 2003). Studies of the influence of SGE on the isolated perfused rat heart revealed a significant decrease in left ventricular pressure without affecting the coronary flow (Rajská *et al.*, 2007). Moreover, application of 3 µg of proteins from the SGE, corresponding approximately to one salivary gland equivalent, stimulated the cardiac sarcolemmal Na,K-ATPase (Takáč *et al.*, 2006). To function, this enzyme utilizes energy derived from hydrolysis of ATP. Therefore, in the present study we focused our interest on a more detailed characterization of the dose-response relationship of SGE on ATP-binding properties on Na,K-ATPase by investigating the enzyme's kinetic behavior in the presence of increasing concentrations of ATP. Since the activity of the enzyme and the number of copies of the pump in vessels are very low in comparison with other tissues such as the heart, we used cardiac sarcolemmal membranes for this study.

## Materials and methods

### Horse fly collection

Horse flies were collected in selected sites of south-western and western Slovakia using Manitoba traps. The effectiveness of the traps was improved by application of CO_2_. Collections were performed during optimal weather conditions (sunny days, temperature 24–28°C, no wind) from May until the end of August. The collecting day started at 9:00 a.m. and finished at 5:00 p.m. Approximately, 100–150 female horse flies per trap were collected each day of trapping, resulting in a total of 1,300 specimens.

### Salivary gland sample preparation and purification

Horse flies were transported to the laboratory alive and then immediately processed. Prior to the dissection of salivary glands, horse-flies were immobilized for a few minutes by placing them at 4 °C conditions. The salivary glands were dissected under a microscope and transferred to Eppendorf vials with cooled PBS buffer (10 mmol·l^–1^ phosphate buffer and 150 mmol·l^–1^ NaCl, pH = 7.2). Samples were homogenized and centrifuged at 2,500 g for 10 min. The supernatant (referred to as crude SGE) was collected and stored at –70 °C until use.

### Isolation of sarcolemmal membranes

After humane sacrificing of the animals, hearts from Wistar Kyoto rats were quickly excised and immediately frozen in liquid nitrogen and stored for further biochemical investigations. Cardiac sarcolemma was prepared from pooled samples of two hearts by the hypotonic shock-NaI treatment method as described previously (Vrbjar *et al.*, 1984). The protein content was assayed by the procedure of Lowry *et al.* (Lowry *et al.*, 1951) using bovine serum albumin as a standard. All experiments were approved by the Veterinary Council of the Slovak Republic (Decree No. 289, part 139, July 9th 2003) and they conform to Principles of Laboratory Animal Care (NIH publication 83-25, revised 1985).

### Kinetics of Na,K-ATPase

The substrate kinetics of Na,K-ATPase were assessed by measuring the hydrolysis of ATP by 30 µg sarcolemmal proteins at 37 °C in the presence of increasing concentrations of ATP (0.08–4.0 mmol/l). Assays were undertaken in a total volume of 0.5 ml medium containing 50 mmol·l^–1^ Imidazole (pH 7.4), 4 mmol·l^–1^ MgCl_2_, 10 mmol·l^–1^ KCl and 100 mmol·l^–1^ NaCl. After 15 min of pre-incubation in the substrate-free medium, the reaction was started by addition of ATP and 15 min later it was terminated by 1 ml of 12% w/v of trichloroacetic acid. Verification of the time dependence of ATP-hydrolysis showed that up to 20 min ATP hydrolysis was linear throughout the ATP concentration range. The liberated inorganic phosphorus was determined according to Taussky and Shorr (1953). In order to determine Na,K-ATPase activity, ATP hydrolysis that occurred in the presence of Mg^2+^ only was subtracted.

### Influence of SGE

The influence of crude SGE from *Hybomitra bimaculata* on the function of the Na,K-ATPase was tested *in vitro* by addition of various amounts of SGE (1.0–6.5 µg proteins) to 30 µg of sarcolemmal proteins. The SGE-quantity was expressed as the amount of applied proteins from the extract.

### Data processing

Kinetic parameters were evaluated by direct nonlinear regression of the data. All results were expressed as mean ± SEM. Significances of differences among the groups were determined using ANOVA, Student-Newman-Keul's test. A value of *p*<0.05 was regarded as significant.

## Results

The effect of SGE from *Hybomitra bimaculata* on the functional properties of Na,K-ATPase in sarcolemmal membrane fraction was investigated *in vitro* using SGE concentrations of 1.0–6.5 µg total protein.

**Figure 1:**
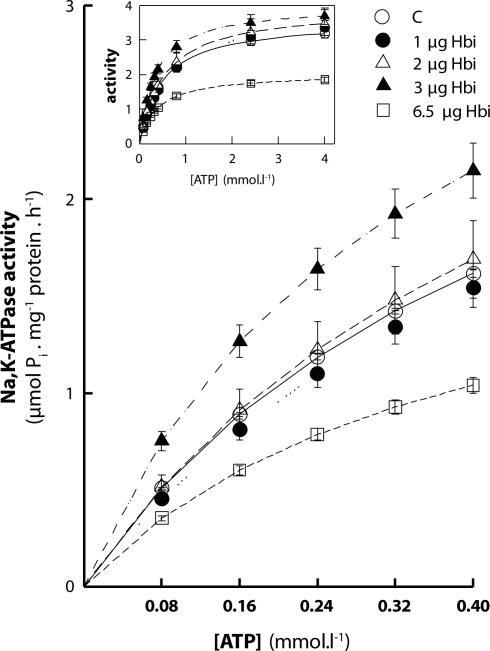
*In vitro* effect of salivary gland extract (SGE) from *Hybomitra bimaculata* (Hbi) on cardiac Na,K-ATPase activity. SGE is expressed as total protein concentration.

In the range of 1–2 µg SGE did not affect significantly Na,K-ATPase activity at any of the ATP concentrations applied (Figure 1). The kinetic parameters of the enzyme V_max_ and K_M_ did not differ significantly from the controls which were measured in the absence of SGE (Figsures 2 and 3).

**Figure 2:**
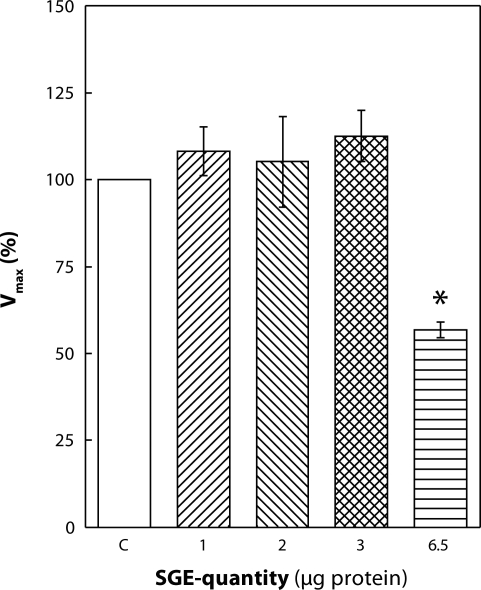
*In vitro* effect of salivary gland extract (SGE) of *Hybomitra bimaculata* on V_max_ values of Na,K-ATPase from rat heart. SGE is expressed as total protein concentration. Data show means ± SEM of 3 estimations, *p<0.05.

**Figure 3:**
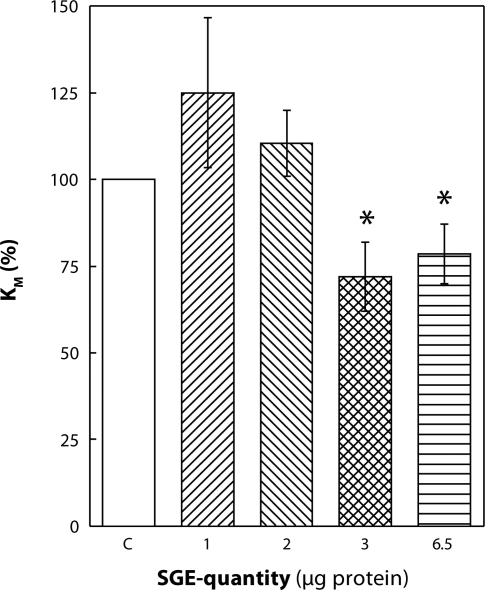
*In vitro* effect of salivary gland extract (SGE) of *Hybomitra bimaculata* on K_M_ values of Na,K-ATPase from rat heart. SGE is expressed as total protein concentration. Data show means ± SEM of 3 estimations, *p<0.05.

Increase in the amount of SGE to 3 µg induced stimulation of Na,K-ATPase at all ATP concentrations applied. The relative increase in enzyme activity was higher at lower concentrations of ATP. At 0.08 mmol·l^–1^ ATP, the stimulation represented 47%. With increasing concentrations of ATP, the effect decreased stepwise and at 4 mmol·l^–1^ ATP the stimulation represented only 16% (Figure 1). By evaluating kinetic parameters, we observed a significant decrease in K_M_ value by 28% (Figure 3) although V_max_ was unchanged (Figure 2).

Addition of 6.5 µg SGE into the reaction medium was followed by an opposite effect to that observed at lower concentrations. In the presence of this high amount of SGE, Na,K-ATPase was inhibited at all concentrations of ATP applied (Figure 1). With increasing concentration of ATP, the inhibitory effect of SGE gradually increased from 30% at 0.08 mmol·l^–1^ to 42% at 4 mmol·l^–1^ ATP. Kinetic parameters presented in Figures 2 and 3 show a statistically significant decrease in V_max_ by 43% along with a significant lowering of K_M_ by 21%. The K_M_ value is lowered mainly as a result of higher inhibition of the enzyme at 0.8–4 mmol·l^–1^ ATP concentration, suggesting reduced ATP-utilization by the enzyme, especially at higher concentrations of the substrate.

## Discussion

To overcome the defensive reaction of vertebrates during blood sucking, hematophagous insects usually inject into the site of injury specific compounds with anticoagulant (Kazimírová *et al.*, 2001; Kazimírová *et al.*, 2002; Xu *et al.*, 2008), immunoregulatory (Yan *et al.*, 2008; Zhao *et al.*, 2009) and vasodilatory activities (Lerner *et al.* 1991, Ribeiro, 1992; Ribeiro 7 Nussenzveig, 1993).

Previous studies dealing with *Hybomitra bimaculata* have shown that horse flies also secrete compounds with vasodilating activity, as documented by the relaxation response of the rat femoral artery. For example application of the equivalent to one salivary gland of *Hybomitra bimaculata* induced an increase in relaxation of 45% (Takáč *et al.*, 2006).

On studying the behavior of Na,K-ATPase as a system involved in the regulation of relaxation-contraction processes in conditions when the enzyme was subjected to the presence of various amounts of salivary gland extract from the horse fly *Hybomitra bimaculata*, we observed various effects of SGE depending on the ratio between the proteins from SGE and the sarcolemmal proteins from the host tissue.

Induction of any effect on Na,K-ATPase requires a certain ratio of SGE to sarcolemmal proteins in the host tissue as demonstrated by unchanged Na,K-ATPase activities in the presence of 1–2 µg SGE proteins.

Application of a higher ratio of SGE proteins (3 µg SGE proteins *vs*. 30 µg of sarcolemmal proteins) induced a stimulation in Na,K-ATPase activity especially at low ATP concentrations. This effect may be induced by a component of SGE which increases the affinity of the ATP-binding site in the Na,K-ATPase molecule, as suggested by a significant decrease of the K_M_ value, while the V_max_ remained unchanged. As a consequence of improved affinity to ATP, the enzyme is able utilize better its energy substrate, resulting in higher extrusion of intracellular Na^+^ and thus in enhancement of relaxation and decline of contraction.

In contrast, application of the highest ratio of SGE proteins in our experiment (6.5 µg SGE proteins *vs*. 30 µg of sarcolemmal proteins) resulted in an inhibitory effect on Na,K-ATPase. Due to the different mechanisms of action of SGE on Na,K-ATPase troughout the ATP concentration range applied depending on the ratio of SGE proteins to sarcolemmal proteins, the most probable explanation seems to be the possibility that another component from the SGE inhibits the Na,K-ATPase-substrate complex when the ATP is already bound to the enzyme. As the inhibition occurred at relatively high concentrations of SGE compared with sarcolemmal proteins, the inhibitory effect is probably localized to the very small area around the injury of the host tissue. In this small area the SGE-induced inhibition of Na,K-ATPase impairs the extrusion of intracellular sodium ions, resulting in decline of relaxation and enhancement of contraction. This finding is in agreement with observations that reduction of the transmembrane Na^+^ gradient substantially augments contractions in vascular smooth muscle by blocking Na,K-ATPase (Ashida & Blaustein, 1987; Borin *et al.*, 1994).

Due to the fact that the same role in the mechanism of relaxation was ascribed to Na,K-ATPase in cardiac and also in vascular tissue, it may be assumed that the enzyme from cardiac sarcolemma may be used as an adequate model for the enzyme function also in vascular tissue. Based on our findings, we may hypothesize that directly at the site where the horse fly is biting, some compounds from its saliva cause a contraction of muscle cells induced by inhibition of Na,K-ATPase, thus fixing the vascular tissue of the host during the process of bloodsucking. In the vicinity of this site, other component from the insect's saliva improve the relaxation by stimulation of Na,K-ATPase in host tissue, providing thus a better blood supply in the close vicinity. In remote parts of the host tissue, the saliva from *Hybomitra bimaculata* does not induce any significant effect on Na,K-ATPase.
